# Electronic Symptom Reporting Between Patient and Provider for Improved Health Care Service Quality: A Systematic Review of Randomized Controlled Trials. Part 2: Methodological Quality and Effects

**DOI:** 10.2196/jmir.2216

**Published:** 2012-10-03

**Authors:** Monika Alise Johansen, Gro K Rosvold Berntsen, Tibor Schuster, Eva Henriksen, Alexander Horsch

**Affiliations:** ^1^Norwegian Centre for Integrated Care and TelemedicineUniversity Hospital of North NorwayTromsøNorway; ^2^Institute of Medical Statistics and EpidemiologyTechnische Universität MünchenMunichGermany; ^3^Department of Telemedicine and e-HealthInstitute of Clinical MedicineUniversity of TromsøTromsøNorway

**Keywords:** Electronic symptom reporting, physician-patient relationship, patient participation, shared decision making, review, consultation, monitoring, self-management, bias

## Abstract

**Background:**

We conducted in two parts a systematic review of randomized controlled trials (RCTs) on electronic symptom reporting between patients and providers to improve health care service quality. Part 1 reviewed the typology of patient groups, health service innovations, and research targets. Four innovation categories were identified: consultation support, monitoring with clinician support, self-management with clinician support, and therapy.

**Objective:**

To assess the methodological quality of the RCTs, and summarize effects and benefits from the methodologically best studies.

**Methods:**

We searched Medline, EMBASE, PsycINFO, Cochrane Central Register of Controlled Trials, and IEEE Xplore for original studies presented in English-language articles between 1990 and November 2011. Risk of bias and feasibility were judged according to the Cochrane recommendation, and theoretical evidence and preclinical testing were evaluated according to the Framework for Design and Evaluation of Complex Interventions to Improve Health. Three authors assessed the risk of bias and two authors extracted the effect data independently. Disagreement regarding bias assessment, extraction, and interpretation of results were resolved by consensus discussions.

**Results:**

Of 642 records identified, we included 32 articles representing 29 studies. No articles fulfilled all quality requirements. All interventions were feasible to implement in a real-life setting, and theoretical evidence was provided for almost all studies. However, preclinical testing was reported in only a third of the articles. We judged three-quarters of the articles to have low risk for random sequence allocation and approximately half of the articles to have low risk for the following biases: allocation concealment, incomplete outcome data, and selective reporting. Slightly more than one fifth of the articles were judged as low risk for blinding of outcome assessment. Only 1 article had low risk of bias for blinding of participants and personnel. We excluded 12 articles showing high risk or unclear risk for both selective reporting and blinding of outcome assessment from the effect assessment. The authors’ hypothesis was confirmed for 13 (65%) of the 20 remaining articles. Articles on self-management support were of higher quality, allowing us to assess effects in a larger proportion of studies. All except one self-management interventions were equally effective to or better than the control option. The self-management articles document substantial benefits for patients, and partly also for health professionals and the health care system.

**Conclusion:**

Electronic symptom reporting between patients and providers is an exciting area of development for health services. However, the research generally is of low quality. The field would benefit from increased focus on methods for conducting and reporting RCTs. It appears particularly important to improve blinding of outcome assessment and to precisely define primary outcomes to avoid selective reporting. Supporting self-management seems to be especially promising, but consultation support also shows encouraging results.

## Introduction

This paper presents the second part of a comprehensive review of randomized controlled trials (RCTs) focusing on electronic communication between patient and provider to improve health care service quality. This patient-provider partnership is defined by patients or parents reporting symptoms or health information electronically [1]. The patient reports to health care personnel, an institution, or a system, where the receiver processes and interprets the data and provides feedback to the patient [1]. The general purpose is improved health care service quality and efficiency, for example, by improving or avoiding a consultation [1].

Part 1 of this review identified the following typology of the field in terms of [2] patient groups, health service innovations, and research targets:

- Five specific patient groups mainly based on the International Classification of Primary Care (ICPC) definitions [3]: cancer, respiratory and lung diseases, cardiovascular diseases, psychiatry, and diabetes.
- Four health service innovation categories: consultation support, monitoring with clinician support, self-management with clinician support, and therapy.
- Research targets: consultation support studies primarily aimed to improve patient-centered care and secondarily to provide health benefits. Monitoring studies focused on health benefits, patient-centeredness outcomes, and reduced health care costs. Self-management studies mainly aimed for health benefits and secondarily patient-centered outcomes.

This part of the review looked into the methodological quality on the RCTs, and summarized effects and benefits of electronic symptom reporting of the methodologically best RCTs.

### Effects and Benefits of Electronic Symptom Reporting

It is possible to achieve effects of electronic symptom reporting at the health care professional, health care system, and patient levels.

At the *health care professional level*, electronic symptom reporting might support the diagnostic process, and thus also make better use of the health professional’s time. Determining the patient’s main problem or concern is often demanding for the physician [4]. The way in which patients present their problems, and the sequence, importance, and severity of symptoms influence the physician’s interpretation. Likewise, studies of interview styles show that physicians elicit only about 50% of the medical information considered important in a consultation [5]. Health care professionals may also be challenged by patients’ difficulties in correctly remembering symptom levels beyond the past several days [6] and older patients’ omission of many symptoms [7] during a consultation. On the other hand, we know that people in general report a higher number of and more serious symptoms when using computer-mediated communication than in face-to-face encounters or phone conversations [8] (p. 28-29).

At the *health care system level*, time and money might be saved [9]. Trials of electronic symptom reporting suggest that it may be possible to substitute about one-third or more of face-to-face consultations in primary care settings [10,11]. It is probably also possible to reduce the number of consultations in specialist care. Internationally, up to 24% of surgeries are cancelled the same day as they are scheduled [12-14], which is a major expense for health care systems [13]. Patient information might be outdated, inadequate, or even wrong at the time of surgery [15,16], and nearly half of the cancellations could have been avoided with an adequate patient information review and update [13].

At the *patient level*, it is possible to improve documentation of key variables that affect service quality and safety [17]. Patients embrace the idea of reporting symptoms electronically before their visit to the doctor [18-21] and believe it will improve the quality of care and effectiveness during the encounter [19,20]. Wald et al demonstrated that 70% of 2027 patients actually submitted symptom information before consultation, and that patients felt more prepared for the visit and that their provider had more accurate information about them [22]. These findings give rise to the assumption that electronic symptom reporting might be a useful tool to strengthen patient empowerment. Patients who report symptoms electronically prior to a consultation are given a chance to convey their problems in a less-stressful situation. This may result in patients having a preformed clear and concise understanding of their own clinical problems, while at the same time it provides updated patient information and documentation that can be saved in the electronic patient record. This may improve the diagnostic process and result in better patient management and care planning.

However, our preliminary screening found that studies in the field typically are small in terms of number of patients involved and are best described as feasibility studies [1]. Many of the studies focused on technologies rather than health effects, and most of them seem to have been underpowered to document clinical effects or specific benefits for health care professionals, health care systems, or patients [1]. No systematic review has yet addressed this topic, to the best of our knowledge, which makes it difficult for innovators and researchers to assess which of these choices are most promising and have the strongest potential for development on a larger scale.

### Methodological Quality

We wanted to limit our work to the most mature stage of a complex intervention before implementation, the RCTs [23,24]. Unfortunately, the overall quality of RCT reporting is not always satisfactory [25]. Studies of low methodological quality typically tend to report better treatment effects than do studies of high quality [26-28]. Despite the development of guidelines to improve RCT reporting [29], it is still necessary to assess the methodological quality of RCTs, in our case for studies on electronic symptom reporting.

### Objectives

The overall aim of this review was to systematically assemble the knowledge gained from RCTs focusing on electronic communication between patient and provider to improve health care service quality.

The objective for this second part of the review was to (1) assess the methodological quality of the RCTs identified in the first part of the review, and (2) summarize effects and benefits of electronic symptom reporting from data published in the methodologically best RCT articles. The benefits will be presented with regard to patients, health care professionals, and health care systems.

## Methods

The review in general followed the Preferred Reporting Items for Systematic Reviews and Meta-analyses (PRISMA) recommendations [30]. To further improve the quality, we consulted the Cochrane handbook [31] for data extraction and assessment of methodological quality. The group conducting the review has multidisciplinary background, including experience in medical and epidemiological research (GB, AH, TS), RCT methodology and statistics (TS, GB, AH), telemedicine and medical informatics (MAJ, EH, AH, TS), theoretical knowledge of electronic symptom reporting (MAJ, EH), and experience from earlier review work (AH, GB, TS).

### Inclusion and Exclusion Criteria

We included original RCTs if patients or parents reported recent symptoms or health information electronically, either to clinical health care personnel or to a system, where the receiver processed and interpreted the data for health care purposes and provided feedback. Feedback did not need to be given electronically. The focus was on asynchronous systems that can be established within the health care system. If the control group reported symptoms or health information, this information was not received by the health care professional or system. For a detailed description of the inclusion and exclusion criteria, see part 1 of the review [2].

### Search Methods for Identification of Studies

We searched Medline, EMBASE, PsycINFO, the Cochrane Central Register of Controlled Trials, and IEEE Xplore to retrieve RCTs about human medicine presented in the English language, published from 1990 to November 2011. For a detailed description of the search methods, see part 1 of the review [2].

### Selection of Studies and Data Extraction

Studies (abstract and full text) were selected independently by two authors (MAJ and EH), and all disagreements were resolved by consensus discussions. We extracted 84 variables from each included article, guided by the Cochrane data collection checklist [31] (Table 7.3.a in the Cochrane handbook). Variables defined as especially relevant for this specific review were included.

Extracted variables focused on the assessment of methodological quality, including evaluation of the risk of bias in the results, and outcome measures and results relevant to electronic symptom reporting. A full presentation of the extracted variables and the citations can be found on the website of the Norwegian Centre for Integrated Care and Telemedicine [32]. A comprehensive description regarding selection of studies and data extraction and management is given in part 1 of the review [2].

### Assessment of Methodological Quality

We assessed the methodological quality of each article, including risk of bias and three additional variables reflecting feasibility, theoretical evidence, and preclinical testing.

Risk of bias was assessed according to Cochrane’s recommended domain-based evaluation, the criteria for judging risk of bias [31] (Table 8.5.d in the Cochrane handbook), and judged as low risk, unclear risk, or high risk.

Theoretical evidence and preclinical testing are both recommended as part of a framework for the design and evaluation of complex interventions to improve health [23,24]. An RCT should rest firmly on both a theoretical foundation and practical testing of how that theory can be applied in a specific context. Without such prior exploration, a nonsignificant finding may result from several causes that have nothing to do with the intervention itself, leading to a wrong conclusion. Thus, we included three additional quality assessment variables, referring to (1) whether implementing the intervention as planned is feasible or likely in a real-life setting [31], (2) theoretical evidence that the intervention might have the desired effects, and (3) preclinical testing, referring to the process of operationalizing theories through pilot trials and feasibility or acceptability testing [23,24].

The “assessment of the overall risk of bias involves consideration of the relative importance of different [bias] domains” and the review author’s judgments “about which domains are most important in the current review” [31] (chapter 8.7). Given the nature of telemedicine and eHealth innovations, blinding of participants and personnel is extremely challenging. We thus did not consider this bias to be crucial to the quality judgment of the articles. As it is difficult to blind participants and personnel in electronic symptom reporting settings, we attached more importance to the blinding of outcome assessment. This kind of blinding is both possible and realistic to achieve, and might affect the study result. We also considered selective reporting to be important, since it indicates post hoc selection of a subset of the original analyses performed [31] (chapter 8.14.1), while typically omitting the negative analyses answering the original research question. Selective reporting thus causes publication bias, as negative results tend to be left unreported, and spurious random findings are highlighted instead. We considered a low risk of bias for selective reporting and for blinding of outcome assessment to be the best indicators for identification of studies with high methodological quality.

Incomplete outcome data refers both to attrition and to exclusion of participants through as-treated or other subgroup analyses. When assessing selective reporting, we accepted that a primary outcome variable could be represented through a group of well-defined measures, as long as authors reported all measures and time points properly and completely in the results section. On the other hand, we assessed studies as having a high risk for selective reporting if we found any incongruence between the published protocol and the reported primary results, or if the variable used to make power calculations was not part of the reported primary outcome measures. We applied the same logic if the authors’ main conclusions did not rely on previously defined outcome parameters and therefore had to be regarded as limitedly interpretable post hoc findings.

The blinding of outcome assessment risk of bias was judged as high if there was no information indicating involvement of any independent personnel for assessment of outcome other than those performing the intervention. If the patients were the outcome assessors for the primary outcome, and all the patients had access to a common online discussion page, we regarded this as having knowledge about which intervention they and other patients received.

The Cochrane criteria for the unclear risk judgment is primarily defined as “insufficient information to permit judgment of ‘low risk’ or ‘high risk’” [31] (Table 8.5.d in the Cochrane handbook). This means that everything in the unclear category might be of good or bad quality. However, the fact that the author did not report satisfactorily for us to make a judgment is in itself a bias, which is why we combined the high and unclear category in the analysis of the total bias results.

Three of the authors (TS, GB, MAJ) assessed the risk of bias independently. In all cases of disagreement, a discussion took place until consensus was achieved.

### Effects of Electronic Symptom Reporting

We agree that it is not acceptable to “present analyses and interpretations based on all studies, ignoring flaws identified during the assessment of risk of bias” [31] (chapter 8.8.1). Thus, we excluded articles found to be at high risk or unclear risk for both selective reporting and blinding of outcome assessors from the subsequent analysis of reported effects.

For all other articles, we extracted the primary outcome and present it in an effect table according to the article’s health service innovation category. Some articles defined more than one outcome variable as their primary outcome. In these cases, we chose the first variable presented in the article’s text to be included in our effect table.

Since only a few of these RCTs had a follow-up after the intervention, we chose the immediate postintervention outcome when extracting effects. We calculated within- and between-group pre- to postintervention differences and report the extracted *P *value for the between-group difference. Studies were defined as either equivalence studies (authors hypothesized that the study arms would be equivalent in terms of the effect measure) or as superiority studies (authors hypothesized that one arm would be superior to the other or others in terms of the chosen effect measure). If the authors’ hypothesis was confirmed, we classified the study as positive; otherwise it was negative. Two of the authors (MJ and GB) extracted the effect data independently. In case of disagreement regarding what to extract and how to interpret the results, a discussion to reach consensus was reached.

In addition to the primary outcome effects, other extracted results from the articles with acceptable quality are reported for each health service innovation according to who might benefit: patients, health professionals, or the health care system. The reporting makes use of the Institute of Medicine (IOM) definitions stating that health care should be safe, effective in terms of health benefits, patient centered, timely, efficient (reduced time, reduced health care costs for the health system, and resource utilization of the health professional), and equitable [33]. The extracted outcome variables are based on the Cochrane recommendations, in addition to a set of variables that we developed. The cross-link between who benefits, the extracted outcome variables, and the areas of health service quality defined by IOM is presented in Table 1 in part 1 of the review [2].

## Results

### Main Background Data

Of 642 records identified and 444 abstracts reviewed, 32 articles presenting 29 studies were included [34-65] (see Figure 1 in part 1 [2]). The 32 articles were published from 2002 to 2011, with 24 of them being published in the last 5 years; 27 studies were conducted in Western countries, 12 of these in the United States.

All except 2 studies were designed as parallel studies with random allocation of patients. Of the parallel studies, 4 had three arms [38,47,59,61] and the others had two. A total of 2 studies were based on cluster randomization, 1 on randomized primary care practices [63,64], and 1 on clinics [39]. All studies focused on both genders, and the studies included on average 60% females (varying from 37.5% to 93%).

### Methodological Quality

Even if we accept that patients and personnel were not blinded, no articles met all the quality requirements, and many articles satisfied few methodological quality criteria (Table 1, low risk or yes). Only 2 of the articles, Bergström et al [60] and Schwarz et al [54], had a low risk for all types of bias except blinding of participants and personnel. However, they did not fulfill the preclinical testing requirements. For 3 of the articles, Boyes et al [35], Santamore et al [53], and Williams et al [64], we found no types of bias to be at low risk.

All articles had interventions that could be implemented as planned in a real-life setting. Thus, this aspect is not included in Table 1. Theoretical evidence was provided in almost all articles. However, preclinical testing was properly provided for only about a third of the articles.

The quality assessment with regard to random sequence allocation was the bias domain with the best results, with three-quarters of the articles judged to have low risk. For only about half of the articles, the risk of bias for allocation concealment, incomplete outcome data, and selective reporting was judged to be low risk. With regard to selective reporting, 25% of the articles used several primary outcomes, and 28% had not defined or remained unclear regarding the primary outcome (Tables 4-7 in part 1, [2]).

For barely more than one-fifth of the articles, we judged the blinding of outcome assessment bias to be low risk. High risk of bias due to inadequate concealment of the allocated intervention from participants and personnel during the study is very challenging in telemedicine and eHealth research. This was, not surprisingly, achieved for only 1 of the included articles, Yardley et al [56].

### Effects of Electronic Symptom Reporting

We excluded 12 articles assigned a high risk or unclear risk for both selective reporting and blinding of outcome assessments from the following effect report. We excluded 3 consultation support articles: Berry et al [34], Boyes et al [35], and Stevens et al [39]. We also excluded 8 monitoring articles (representing 6 studies): Chan et al [42], Chan et al [43], Jan et al [45], Kearney et al [41], Lewis et al (quality of life study) [50], Nguyen et al [51], Prabhakaran et al [46], and Santamore et al [53]. Only the secondary analysis of 1 self-management study was excluded (Williams et al [64]).

The reported effects (Table 2, Table 3, Table 4, and Table 5) show that the authors’ hypothesis was confirmed in 13 (65%) of the 20 remaining articles. Interpreting the hypothesis as negative (no) or positive (yes) for primary outcome depended on whether the intervention hypothesis relative to the control condition was stated as equivalent or superior. We considered 4 of the studies to be equivalence studies, in all of which the authors’ hypothesis was confirmed.

### Overall Picture of Evidence

Multimedia Appendix 1 shows that the 20 RCTs with acceptable quality included a total of 3991 patients (ie, 200 patients on average per study). The average number of patients per RCT per combination of patient group and health innovation category ranged from 40 (chronic obstructive pulmonary disease monitoring) to 886 (diabetes self-management). The average per innovation category is comparable for consultation support (181), monitoring (162), and self-management (249), while it is much smaller for therapy (55). Evidence appears most advanced in the self-management category, with a total of 9 RCTs including more than half of the total number of patients.

#### Main Research Focuses and Study Results

Table 6 gives an overview of the main research focuses and study results for the 20 articles included in the effect review. *Study *is now equivalent to *article*, since none of these studies were reported in more than 1 article after exclusion by quality. Self-management appears to be the most promising health service innovation category, since the hypothesis was confirmed for 8 of the 9 studies.

In the monitoring category, 2 of the asthma studies confirmed their hypothesis, while we lack positive results for chronic obstructive pulmonary disease and cardiovascular monitoring. Also, the hypothesis was confirmed in 2 studies on consultation support in the cancer patient group.

**Table 1:** Judgments of methodological quality in the reviewed randomized controlled trials of electronic symptom reporting^a^.

Article	Theoretical evidence/ preclinical testing	Random sequence generation^b^	Allocation concealment^b^	Blinding of participants and personnel^b,c^	Blinding of outcome assessment^b,d^	Incomplete outcome data^b^	Selective reporting^b,d^
Berger et al [59]	yes/yes	+	+	–	–	+	+
Bergström et al [60]	yes/unclear	+	+	–	+	+	+
Berry et al [34]	yes/yes	+	+	–	–	+	–
Boyes et al [35]	yes/no	○	○	–	–	–	–
Carrasco et al [52]	yes/no	+	–	–	–	+	+
Chan et al [42]	yes/no	+	○	–	–	○	–
Chan et al [43]	yes/yes	+	○	–	–	–	–
DeVito Dabbs et al [55]	yes/yes	+	–	–	–	+	+
Glasgow et al [63]	yes/yes	+	+	–	–	○	+
Guendelman et al [44]	yes/yes	○	+	–	–	+	+
Jan et al [45]	yes/no	○	+	–	–	+	–
Kearney et al [41]	yes/yes	+	+	–	–	–	–
Leveille et al [40]	yes/no	○	○	–	–	+	+
Lewis et al [49] (hospitalization)	yes/unclear	+	+	–	+	○	–
Lewis et al [50] (quality of life)	unclear/unclear	+	+	–	–	○	–
Nguyen et al [58]	yes/yes	+	+	–	–	–	+
Nguyen et al [51]	yes/no	+	+	–	–	+	–
Oerlemans et al [62]	yes/no	+	–	–	–	+	+
Prabhakaran et al [46]	no/no	+	+	–	–	+	–
Rasmussen et al [47]	yes/no	–	+	–	–	○	+
Ruland et al [36]	yes/yes	○	○	–	○	○	+
Ruland et al [37]	yes/yes	+	–	–	+	+	+
Santamore et al [53]	no/no	○	○	–	–	○	–
Schwarz et al [54]	yes/no	+	+	–	+	+	+
Stevens et al [39]	yes/no	+	○	–	–	–	–
van der Meer et al [57]	yes/unclear	+	+	–	–	+	+
Velikova et al [38]	yes/no	+	○	–	+	+	+
Vernmark et al [61]	yes/no	+	○	–	+	+	+
Wagner et al [65]	yes/no	+	○	–	–	+	+
Willems et al [48]	yes/no	+	–	–	–	+	+
Williams et al [64]	no/no	○	○	–	–	○	○
Yardley et al 2010 [56]	yes/no	+	+	+	+	–	○

^a ^Articles were identified in a comprehensive search in Medline, EMBASE, PsycINFO, Cochrane Central Register of Controlled Trials, and IEEE Xplore from 1990 to November 2011, and were published in the time period 2002–2011.

^b ^+ = low risk, ○ = unclear risk, and – = high risk.

^c ^Blinding of participants and personnel is extremely challenging in telemedicine and eHealth innovations. We thus did not consider this bias to be crucial to the quality judgment of the articles.

^d ^We considered a low risk of bias for selective reporting and for blinding of outcome assessment to be the best indicators for identification of studies with high methodological quality.

**Table 2:** Effects reported in consultation support randomized controlled trials of acceptable quality of electronic symptom reporting, in alphabetic order of first author^a^.

Article	Primary outcome	Intervention hypothesis relative to control	Number at randomization^b^			Within-group pre–post change^b^			Between-group pre–post change^b,c^		Hypothesis confirmed?
			C	I1	I2	C	I1	I2	C-I1	C-I2	
Leveille et al [40]	Number of patients discussing chronic condition with physician during consultation	Superior	120	121	NA^d^	–80.5%	–84.9%	NA	4.4% *P *= .37	NA	No
Ruland et al [36]	Congruence (weighted) between patients’ preconsultation-reported health issues and issues discussed in consultation	Superior	25	27	NA	–12.8	–33	NA	20.2 *P *< .01	NA	Yes
Ruland et al [37]	Number of patients’ symptoms and problems addressed by physicians as documented in inpatients’ records	Superior	70	75	NA	–7.9	–10	NA	2.1 *P *< .001	NA	Yes
Velikova et al [38]	Health-related quality of life, functional assessment of cancer	Superior	72	144	70	NR^e^	NR	NR	–0.02 *P *= .3	NR	No

^a ^Articles were identified in a comprehensive search in Medline, EMBASE, PsycINFO, Cochrane Central Register of Controlled Trials, and IEEE Xplore from 1990 to November 2011, and were published in the time period 2002–2011.

^b ^C = control group, I1 = intervention group 1, I2 = intervention group 2.

^c^ *P *values for difference between groups.

^d ^Not applicable.

^e ^Not reported.

**Table 3:** Effects reported in monitoring with clinical support randomized controlled trials of acceptable quality of electronic symptom reporting, in alphabetic order of first author^a^.

Article	Primary outcome	Intervention hypothesis relative to control	Number at randomization^b^			Within-group pre–post change^b^			Between-group pre–post change^b,c^		Hypothesis confirmed?
			C	I1	I2	C	I1	I2	C-I1	C-I2	
Carrasco et al [52]	Number of patients exhibiting poor hypertension control	Superior	143	142	NA^d^	64.3%	68.3%	NA	–4.0% *P *= .47	NA	No
Guendelman et al [44]	Patients experiencing limitations in activity due to asthma last 14 days	Superior	68	66	NA	25%	35%	NA	–9% *P *= .03	NA	Yes
Lewis et al [49]	Number of hospitalizations	Superior	20	20	NA	–7	–4	NA	–3 *P *= .16	NA	No
Rasmussen et al [47]	FEV_1_^e^	Superior	100	100	100	0.004	0.187	0.035	–0.183 *P *< .001	–0.031 Nonsignificant	I1: Yes
Schwarz et al [54]	Mean number of hospital readmissions in group	Superior	51	51	NA	–0.33	–0.32	NA	–0.01 *P *= .9	NA	No
Willems et al [48]	Asthma-specific quality of life	Superior	54	55	NA	–0.06	–0.29	NA	0.23 *P *= .386	NA	No

^a ^Articles were identified in a comprehensive search in Medline, EMBASE, PsycINFO, Cochrane Central Register of Controlled Trials, and IEEE Xplore from 1990 to November 2011, and were published in the time period 2002–2011.

^b ^C = control group, I1 = intervention group 1, I2 = intervention group 2.

^c^ *P *values for difference between groups.

^d ^Not applicable.

^e ^Forced expiratory volume of air in the first second of expiration.

**Table 4:** Effects reported in self-management with clinical support randomized controlled trials of acceptable quality of electronic symptom reporting, in alphabetic order of first author^a^.

Article	Primary outcome	Intervention hypothesis relative to control	Number at randomization^b^			Within-group pre–post change^b^			Between-group pre–post change^b,c^		Hypothesis confirmed?
			C	I1	I2	C	I1	I2	C-I1	C-I2	
Berger et al [59]	Social Phobia Scale	Equivalence	27	27	27	16.2	16.3	17.9	–0.1 *P *= .90	–1.7 *P *= .90	Yes
Bergström et al [60]	Panic Disorder Severity Scale	Equivalence	60	53	NA^d^	NR^e^	NR	NA	*P *= .95	NA	Yes
DeVito Dabbs et al [55]	Perceived self-care agency (in follow-up after surgery)	Superior	17	17	NA	–15.1	–19.44	NA	4.34 *P *= .003	NA	Yes
Glasgow et al [63]	Number of laboratory procedures completed in accordance with national diabetes guidelines	Superior	28^f^/417	24^f^/469	NA	–0.09	–0.37	NA	0.28 *P *= .001	NA	Yes
Nguyen et al [58] (dyspnea)	Patients’ self-report of dyspnea with activities of daily living, Likert scale	Equivalence	24	26	NA	–4	–2.5	NA	–1.5 *P *= .51	NA	Yes
Oerlemans et al [62]	Cognitive Scale for Functional Bowel disorders	Superior	38	38	NA	1.84	8.99	NA	–7.15 *P *> .05	NA	No
van der Meer et al [57]	Asthma-related quality of life	Superior	99	101	NA	–0.18	–0.56	NA	0.38 *P *< .001	NA	Yes
Vernmark et al [61]	Symptom reduction: Beck Depression Inventory	Equivalence	29	29	30	5.2	9.9	11.9	–4.7 *P *= .06	–6.7 *P *= .002	Yes
Yardley et al [56]^g^	Patient enablement scores, after 4 weeks.	Superior	346	368	NA	2	3	NA	–1 *P *= .03	NA	Yes

^a ^Articles were identified in a comprehensive search in Medline, EMBASE, PsycINFO, Cochrane Central Register of Controlled Trials, and IEEE Xplore from 1990 to November 2011, and were published in the time period 2002–2011.

^b ^C = control group, I1 = intervention group 1, I2 = intervention group 2.

^c^ *P *values for difference between groups.

^d ^Not applicable.

^e ^Not reported.

^f ^Cluster randomized by primary care physicians.

^g ^214 replied with regard to patient enablement, 95 in the intervention group, 119 in control.

**Table 5:** Effects reported in therapy randomized controlled trials of acceptable quality of electronic symptom reporting^a^.

Article	Primary outcome	Intervention hypothesis relative to control	Number at randomization^b^			Within-group pre–post change^b^			Between-group pre–post change^b,c^		Hypothesis confirmed?
			C	I1	I2	C	I1	I2	C-I1	C-I2	
Wagner et al [65]	Intrusion measured by the Impact of Event Scale	Superior	26	29	NA^d^	3.77	11.5	NA	–7.73 *P *< .1	NA	Yes^e^

^a ^Articles were identified in a comprehensive search in Medline, EMBASE, PsycINFO, Cochrane Central Register of Controlled Trials, and IEEE Xplore from 1990 to November 2011, and were published in the time period 2002–2011.

^b ^C = control group, I1 = intervention group 1, I2 = intervention group 2.

^c^ *P *values for difference between groups.

^d ^Not applicable.

^e ^Authors considered *P *< 0.1 to be statistically significant.

**Table 6:** Main research focus and overview of confirmed (+) and not confirmed (–) hypothesis for articles included in effect review of randomized controlled trials of electronic symptom reporting, by health service innovation category and patient group^a^.

Patient group	Consultation support	Monitoring with clinical support	Self-management with clinical support	Therapy
Cancer	3 articles (2 excluded): More symptoms identified and discussed [36,37]++, [38]-	0 articles (1 excluded)		
Respiratory and lung diseases: asthma		3 articles (4 excluded): Improved asthma outcome (symptoms or quality of life): Children: [44]+ Adults: [47]+ Both: [48]-	1 article: Improved asthma-related quality of life [57]+	
Respiratory and lung diseases: COPD^b^		1 article (2 excluded): Reduced health care use [49]-	1 article: Reduced dyspnea [58]+^c^	
Respiratory and lung diseases: other			2 articles: Maximized lung transplant-related health outcomes [55]+ Self-care in management of minor respiratory symptoms [56]+	
Cardiovascular disease		2 articles (1 excluded): Improved hypertension [52]- Reduced health care use and costs [54]-		
Psychiatry	0 articles (1 excluded)		4 articles: Symptom reduction for (1) social phobia [59]+^c^, (2) depression [61]+^c^ More effective cognitive behavior therapy for patients with (1) panic disorder [60]+^c^, (2) irritable bowel syndrome [62]-	1 article: More effective cognitive behavioral therapy for bereaved people with complicated grief [65]+
Diabetes			1 article (1 excluded) Quality of care [63]+	
Mixed	1 article: More symptoms identified and discussed [40]-			
Number of articles excluded due to low quality	3	8	1	0

^a ^Articles were identified in a comprehensive search in Medline, EMBASE, PsycINFO, Cochrane Central Register of Controlled Trials, and IEEE Xplore from 1990 to November 2011, and were published in the time period 2002–2011.

^b ^Chronic obstructive pulmonary disease.

^c ^Hypothesis was to demonstrate equivalence between intervention and control.

#### Effects in Consultation Support

The outcomes were categorized by IOM’s quality domains [33]. In the consultation support category, all studies provided patient-centered care, ensuring that patient-reported symptoms guided the clinical decisions. Except for the study where nurses coached patients [40], symptom reporting was generally conducted while the patient was present at the clinic, and a summary of the reported symptoms was made available to the physician [36-38]. These summaries were found effective in identifying and prompting discussion of troublesome symptoms, which made it possible to focus the conversation on issues relevant to the patient’s problems [36-38].

The electronic symptom reporting systems also showed positive outcomes for patient symptom distress [37], symptom management [37], and health-related quality of life [38]. Patients supported the gathering of symptom information by computerized survey [36,40] and spent a median of 9 minutes reporting [36].

Most clinicians found the summaries useful for identifying problems and providing communication [38], which reduced the need for symptom management support [37]. Benefits for the health care system were mainly that visit duration was similar with and without use of the summaries [38].

The one trial with patient coaching [40] did not show benefits for patients or health professionals regarding detection of symptoms and quality of life, but patients in the intervention group reported that they received significantly more advice about their health and referrals to specialist.

#### Effects in Monitoring With Clinical Support

Only 2 monitoring studies reported benefits for patients, while nearly no benefits for the health system and none for the health professionals were reported. The 2 studies identifying health benefits for the patient focused on asthma outcomes for children [44] and adults [47] respectively. Both studies included a strong self-management element. For the latter study, some side effects for the health care system and patient need to be resolved [47].

All the studies, except 1, that aimed to demonstrate reduced health care costs belong to the monitoring group. However, with one exception, no health care costs or health care system benefits were identified: there was no improvement in total number of home care services or informal social support [54], number of consultations [52], occurrence of emergency room visits [44], hospital or specialist team use [49], number of hospital admissions [44,52,54], or mean costs per patient [66]. However, primary care contacts were reduced for patients with chronic obstructive pulmonary disease [49].

#### Effects in Self-management

Articles on self-management support were of higher quality, allowing a larger proportion of studies to be assessed with respect to effects. All self-management interventions were found equally effective to or better than the control option, with only one exception [62]. Substantial benefits for patients, and partly also for health professionals and health care systems, have been documented in this area.

Patient health benefits were reported for follow-up after lung transplantation [55], improved asthma-related quality of life [57], and reduced dyspnea associated with chronic obstructive pulmonary disease [58]. Patient-centered aspects of diabetes care [63], improved level of enablement through Web-based decision support of minor symptoms [56], and satisfaction [56,58] were also documented. Patient health benefits were documented in the psychiatry category for Internet-delivered treatment for social phobia [59], panic disorder [60], major depression [61], and partly for irritable bowel syndrome, with respect to catastrophizing thoughts [62]. Patients also reported a high level of satisfaction [59].

For health professionals, resource utilization was reported. There was some reduction in the number of physician consultations, due to increased asthma control [57] and when patients used the Web-based decision support system providing tailored advice for minor respiratory symptoms [56]. Regarding the latter study, it is important to take into account that the control group used a webpage consisting of advice previously shown to be effective in reducing the number of consultations [56]. In addition, the Internet-delivered treatment of panic disorder used considerably less therapist time than the cognitive behavioral therapy group treatment [60]. However, therapist time for email therapy for major depression was almost 10 times longer than time for guided self-help [61].

At the health care system level, health care cost benefits were analyzed and reported for Internet treatment of panic disorder, which was nearly 4 times cheaper than group treatment [60].

#### Effects in Therapy

Patients receiving email therapy for complicated grief improved significantly relative to participants in the waiting list condition, and were quite satisfied with the treatment [65]. Only 20% missed face-to-face contact with a therapist, and 85% had positive attitudes toward being treated via the Internet instead of face-to-face [65].

## Discussion

Results are discussed with respect to methodological quality of the included RCTs and the effects and benefits gained from electronic symptom reporting between patient and health care provider.

### Principal Findings on Methodological Quality and Effects or Benefits

Overall, the research field appears to be characterized by a comparably large number of low-quality articles that have serious methodological drawbacks.

In total 25% of the articles had multiple primary outcomes, and 28% had not defined or remained unclear regarding the primary outcome.

We extracted effect data only from articles with acceptable quality, which represented 62.5% of all included articles. About half of the articles in the consultation support and monitoring categories were excluded due to low quality, whereas only 1 of the 10 articles on self-management had to be excluded (1 of 2 articles describing the same study). The study hypotheses were confirmed in 13 of the 20 remaining articles. The hypotheses were confirmed in all 4 equivalence studies.

Overall, articles on self-management support were of a higher quality, allowing a larger proportion of studies to be assessed with respect to effects. All the self-management interventions are equally effective to or better than the control option, with one exception [62]. Substantial benefits for patients, and partly also for health professionals and the health care system, have been documented in this area.

In the monitoring trials, health benefits were identified for asthmatic children [44] and adults [47]. Both of these interventions included self-management elements with computer-tailored feedback. Of the 6 monitoring studies, 5 also addressed health care costs, but with one small exception, no cost benefits were identified.

The cancer studies in consultation support are encouraging, since it was found to provide patient-centered care, ensuring that patient-reported symptoms guided the clinical decisions.

### Interpretation of Results

According to our requirements, seven of eight quality criteria should be fulfilled for a study to be considered methodologically correct (accepting that patients and personnel are not blinded). Unfortunately, none of the included articles received positive scores on all criteria, and many articles met just few of them. This lack of adequate methodology negatively affects the overall quality of the RCTs, as pointed out in other studies and reviews [25-27,67,68].

A total of 9 studies had an unclear primary outcome description [2], and all of these studies were excluded from the review of intervention effects due to low quality. Therefore, it is obvious that the field would benefit from a better definition of primary outcome to raise study quality in general, and to avoid selective reporting in particular.

Of the 9 studies with unclear primary outcome and excluded due to low quality, 6 belong to the monitoring category. None of the self-management studies were excluded (only a secondary analysis article where the primary analysis article still is included), which may reflect that this area is more mature. Self-management has already proven to be quite efficient for many long-term diseases [69,70], including psychiatric conditions [71], and various Internet-based setups for self-management have already been used and evaluated for many years.

The heterogeneity in intervention and research targets limits the possibilities to draw reliable conclusions with respect to the effects. Furthermore, designing, conducting, and reporting high-quality RCTs in this field in general is a great challenge, as they have to deal with complex interventions. The complex interventions include several components acting both independently and interdependently [23], and are thus difficult to analyze. If the result is negative, it is hard to judge whether this is because the trial was inadequately developed or applied, or applied in an inappropriate context, or used an inappropriate study design, especially regarding control groups and outcomes [23]. On the other hand, if the result is positive, there is no guarantee that the results can be generalized to a different context [23], not even within the same patient group. For example, in the study of Rasmussen et al where the “study showed that its use resulted in closer monitoring, immediate feedback, adequate medication, and better compliance and that all these initiatives together produced better asthma control” [47], it might be a challenge to judge how the different components affected each other, and how one can repeat the study expecting to obtain the same positive effect. This implies that even implementing an innovation that has been demonstrated to provide a positive effect requires attention and examination of the effects, rate of uptake, intervention stability, and so on [24]. To improve the uptake and impact of technologies in medical care, a holistic framework based on existing eHealth frameworks has recently been suggested [72]. This approach is aware of the existing interdependencies between technology, human characteristics, and the socioeconomic environment, and may be useful for innovating health care, also in future implementations [72]. Another possible next step in the quality assessment process of evaluating possible health service innovations is to use the Model for Assessment of Telemedicine as a guide. This model assists decision makers, before bringing services into everyday use, in predicting medical, social, economic, and ethical issues related to use of the service [73].

### Limitations and Strengths of the Review

An important strength of this review is that the methodological quality assessment was based on Cochrane’s recommended domain-based evaluation for assessment of risk of bias [31], in addition to including three not commonly used quality assessment variables. These variables focus on the feasibility [31] and the theoretical basis for evaluation of complex interventions such as theoretical evidence and preclinical testing recommended by the Framework for Design and Evaluation of Complex Interventions to Improve Health [23,24]. Another important strength is that the risk of bias assessment was conducted by three independent researchers, spending several working days on consensus discussions. A third strength is that effect variables were extracted by two independent persons. A fourth strength is that we followed Cochrane’s recommendations for identifying the types of bias that are most important for the review. A fifth strength is that we took into account the Cochrane warning not to present effects for all studies, and in this way took seriously the flaws identified during the methodology assessment.

However, when conducting the bias evaluation, we could have split the bias regarding blinding of participants and personnel in two—that is, considering study participants and personnel separately. The reason for this is that some of the trials blinded patients to which intervention they received, but did not blind the involved health care personnel.

Some of the assessed articles had sources of bias outside those specified by the Cochrane Collaboration. An example is multiple end-point criteria and hypotheses without adequate adjustment for multiple statistical tests, which may cause problems with final interpretation of results [74]. Another example is unclear statistical analyses of cluster randomized trials. However, we felt that the specified Cochrane bias criteria were sufficiently detailed to discriminate between high- and low-quality articles, which is why we did not systematically extract and present information about other sources of bias.

Interpretation of evidence depends on many factors and is rarely straightforward [75]. Therefore, the interpretation also depends on the reviewers’ experience and background. However, as we always had two or three authors performing the reviewing subtasks independently, with consensus discussions for resolving disagreements, the results of the review should vouch for a high degree of validity and reliability.

Where 8 of the articles used more than one primary outcome, we decided to extract the first variable presented in the text to use in our effect measurement. All 8 had sufficient methodological quality, and 4 demonstrated a positive effect, while the other 4 did not. However, 3 of the 4 that did not demonstrate a positive effect: the self-management study by Oerlemans et al [62] and the consultation support studies by Leveille et al [40] and Velikova et al [38] included other primary outcomes that were positive and significant. We decided to extract the first variable because it is difficult to evaluate a mix of several outcomes; nevertheless, this may be considered a limitation.

Despite using a very comprehensive search strategy, we have reason to believe that we did not quite succeed in covering the area of psychiatry adequately (see part 1 of the review for a more detailed discussion [2]). If the 6 psychiatry studies had not been included, the self-management category would have been reduced, and been less convincing, and the focus for self-management would mainly have been on respiratory and lung diseases. On the other hand, if we had conducted a search that covered the psychiatry field better, we hypothesize, based on reading the studies from the reference lists, that more studies would have been added to both the self-management and the therapy groups. However, the overall quality and effects of electronic symptom reporting within the field of psychiatry are unclear. Therefore, this field deserves its own future review.

As a result of the heterogeneous outcome data in the studies, a meta-analysis was not possible.

### Future Research

Studies of low quality are typically associated with an overestimation of benefits [26-28]. Consequently, improving the methodological quality in the field is essential, and future reviews are necessary to identify whether the methodological quality is improving. This concerns preclinical testing, allocation concealment, incomplete outcome data, and especially risk of bias introduced by selective reporting and nonblinding of outcome assessments being most relevant for this review. Even the blinding of patients and personnel might be achieved in specific studies.

In addition to better definition of the primary outcome to avoid selective reporting, we also recommend improving the account of how the proposed intervention should work, how the intervention links to the outcome measures, and the use of intention-to-treat analysis. As we rarely found that principles such as patient empowerment and patient-centered care were appropriately taken into consideration, this is also a recommendation for future studies.

We also encourage researchers to carefully consider whether it is necessary to demonstrate that the intervention is superior, or if it is sufficient to demonstrate that it is equivalent, as some of the studies designed as superiority studies would have had positive results if they had been designed as equivalence studies. Examples of such studies are the monitoring study of Willems at al [48] and the self-management study of Oerlemans et al [62].

More than half of the monitoring, self-management, and therapy interventions lasted 4 months or less (see part 1), which might be too brief to achieve the intended effect for long-term conditions, especially for complex interventions where both patient and provider often need some time to get used to the technology. Some of the negative studies may have had too short a time frame for an effect to materialize. For example, in Leveille et al’s study of nurse coaching, 38% of intervention participants had less than 2 weeks between completing the screening survey and their indexed appointment, so a longer intervention period might have led to better outcomes [40]. Appreciating the current evidence, we recommend running pilot trials to determine the time frame for effects to appear, and then designing interventions of the necessary length to better document the effects within the electronic symptom reporting field.

The self-management support trials were very successful and showed the most promising results, and should thus be an important guide for further research.

In the consultation support category, two related questions need to be investigated in future studies: (1) does completion of questionnaires, simply giving patients the opportunity to express how they feel, have a positive effect on patient well-being, regardless of whether the results are fed back to physicians? [35,38], and (2) does completion of assessment schemes prior to consultation result in patients recalling their answers and bringing up more symptoms or problems in the consultations [37,39], even if the physician does not read the reported symptoms? A yes to the second question could perhaps explain why the recognition rates in the group where the physician received the summary after the consultation were higher than recognition rates in usual-care samples [39].

The therapy category comprises innovations where the whole treatment, and all communication between therapists and patients, is conducted exclusively electronically. Unfortunately, we identified only 1 therapy article, so we cannot say anything about the general effects without conducting a new search, using specific psychiatry and Internet therapy-related terminology, as suggested in part 1 of the review [2].

An important contribution to the field would be to identify theoretical models that link the health service innovations, and their various components, with expected effects for patients, health professionals, and the health care system in a way that may support the design of the next generation of studies.

### Implications for Practice

The number of studies within each combination of patient group and health service innovation is too small to draw final conclusions. However, if we look at electronic symptom reporting with regard to health service innovations, consultation support and self-management seem to bear various potential benefits for all stakeholders, at the patient, health care provider, and health care system levels.

Symptom reporting in cancer consultation support seems to require little staff effort to empower the patient, and there is little reason to doubt the accuracy of real-time reported symptom data when compared with average or retrospective ratings [6,76]. Reporting data has proven effective in identifying symptoms and prompting discussion of troublesome symptoms, and it allows for focusing the clinic visit conversation on issues relevant to the patient’s problems [36-38]. Electronically reported data makes it possible to easily create clinical databases from which symptom and quality-of-life data can be retrieved, processed, and used in future consultations, surveillance, or for other epidemiological purposes.

Electronic symptom reporting for consultation support should also hold potential for other health conditions—for example, in raising sensitive issues the patient might find difficult to disclose in a face-to-face setting, such as stigma associated with sexually transmitted diseases or mental health problems [8]. However, in the future we expect more generic (not diagnosis-specific) symptom reporting to support consultations to guide both the patient and the clinician. When the patient conducts the reporting, data might be automatically analyzed to provide diagnostic aid for patients, or links to further reading, to prepare patients and thus facilitate more active participation in the treatment [77]. The health care provider, on the other hand, might prepare for recommended examinations on the basis of patient symptoms [78] and suggest solutions to problems and possible diagnoses based on comparable cases [79-81]. The patient and the clinician might then use the information to make a shared decision [82], with improved quality in terms of knowledge and values [83]. Positive effects for the health system seem possible as well—for example, through better information flow, which may avoid unnecessary allocation of resources, and through substitution of face-to-face consultations [10,11].

The positive effects on patients’ self-management should encourage health care providers to promote future services based on the best practice of these innovations. Some of the self-management studies also point to improved cost effectiveness, as shown by Bergström et al with their Internet treatment for panic disorder, which was nearly 4 times cheaper than the group treatment [60]. Vernmark et al did not formally analyze cost effectiveness, but they reported guided self-help as the clinically most feasible option to implement, whereas individualized email therapy turned out to be more costly. However, both treatment effect sizes are in the range of what can be expected from face-to-face treatments [61], so cost-effective solutions might easily be offered to patients over the Internet, providing a more equitable service regardless of geographic location.

This review’s positive result regarding self-management studies should be seen in light of the comprehensive Cochrane review on interactive health communication applications for people with chronic diseases [84]. These applications combine health information with either social support, decision support, or behavior support. The Cochrane review showed that these applications have a significant positive effect on knowledge, clinical outcomes, continuous behavioral outcomes, and the patient’s feeling of being better socially supported [84].

### Conclusion

Even in the subgroup of RCTs, the research methods in the included trials are of low quality. The field would benefit from an increased focus on methods of conducting and reporting RCTs. It appears particularly important to improve blinding of outcome assessment and to more precisely define the primary outcomes to avoid selective reporting.

Electronic communication between patients and health care providers is an exciting area of development for innovative health services, in line with current policies strengthening patient-centered service delivery models and information and communication technologies to increase efficiency and quality. Supporting self-management seems to be especially promising, but results from consultation support trials are also encouraging.

## Multimedia Appendix 1

Number of patients and articles, and the average number of patients per article, in each combination of patient group and health innovation category.

## Abbreviations

ICPC: International Classification of Primary Care
IOM: Institute of Medicine
PRISMA: Preferred Reporting Items for Systematic Reviews and Meta-analyses
RCT: Randomized Controlled Trial
