# Supplementary material for: Electronic Symptom Reporting Between Patient and Provider for Improved Health Care Service Quality: A Systematic Review of Randomized Controlled Trials. Part 2: Methodological Quality and Effects
Source: J Med Internet Res. 2012 Oct 3;14(5):e126. doi: 10.2196/jmir.2216 (PMC3510713; doi:10.2196/jmir.2216)
Supplement: Supplementary file 1 [file jmir_v14i5e126_app1.pdf]

Number of patients (P), number of articles (A) and average number of patients per article (M) for RCT articles on electronic symptom reporting, presented by health service innovation category and patients groups. COPD=Chronic obstructive pulmonary disease. The articles were identified in a comprehensive search in MEDLINE, Embase, PsycINFO, Cochrane Central Register of Controlled Trials and IEEE Xplore from 1990 to November 2011, and published in time period 2002-2011.

**Patient groups**	**Consultation support**	**Monitoring**	**Self-management**	**Therapy**	**Total**
	P / A / M	P / A / M	P / A / M	P / A / M	**P / A / M**
**Cancer**	*483 / 3 / 161*				**483 / 3 / 161**
**Asthma**		*543 / 3 / 181*	*200 / 1 / 200*		**743 / 4 / 185.8**
**COPD**		*40 / 1 / 40*	*50 / 1 / 50*		**90 / 2 / 45**
**Other lung diseases**			*748 / 2 / 374*		**748 / 2 / 374**
**Cardiovascular diseases**		*387 / 2 / 194*			**387 / 2 / 193.5**
**Psychiatry**			*358 / 4 / 89.5*	*55 / 1 / 55*	**413 / 5 / 82.6**
**Diabetes**			*886 / 1 / 886*		**886 / 1 / 886**
**Mixed**	*241 / 1 / 241*				**241 / 1 / 241**
**Total**	**724 / 4 / 181**	**970 / 6 / 162**	**2242 / 9 / 249.1**	**55 / 1 / 55**	**3991 / 20 / 199.6**
